# Current-Induced Evolving Mechanical Properties, Formation of Defects, and Interfacial Intermetallic Growth in the Interconnects Bonded with Au Wire

**DOI:** 10.1021/acsami.5c03751

**Published:** 2025-06-13

**Authors:** Xiaohong Yuan, Qinlian He, Xiaojing Wang, Jiaheng Zhang, Dapeng Yang, Qinsong Bi, Yuxi Luo, Dengquan Chen, Shanju Zheng, Manal S. Ebaid, Hassan Algadi, Zhanhu Guo

**Affiliations:** † Yunnan Precious Metals Lab, Sino-Platinum Metals Co. Ltd., Kunming 650106, China; ‡ Jiangsu University of Science and Technology, Zhenjiang 212003, China; § Faculty of Material Science and Engineering, Kunming University of Science and Technology, Kunming 650093, China; ∥ Department of Chemistry, College of Science, Northern Border University, Arar 91431, Saudi Arabia; ⊥ Department of Mechanical and Construction Engineering, Northumbria University, Newcastle Upon Tyne NE1 8ST, U.K.; # Department of Electrical Engineering, Faculty of Engineering, Najran University, Najran 11001, Saudi Arabia; ¶ State Key Laboratory of Precious Metal Functional Materials, Kunming 650106, China

**Keywords:** wire bonding, Au−Al, electromigration, polarity effect, IMCs, mechanical properties

## Abstract

Continuously improving chip integration and increasing packaging density increase the risk of performance degradation and electromigration (EM) failure on the bonding interface during electrical transmission. While EM failure, as a time-accumulated failure, is one core challenge of semiconductor reliability and is particularly severe in highly integrated chips. However, the polarity differences existing in commercial devices and the evolving polarity characteristics of microscale bonding interfaces have not been well addressed. Therefore, this study describes the polarity effects of EM in a commercial chip-end Au–Al system and reveals the evolution of intermetallic compound (IMC) growth at bonding interfaces under high-density currents. An EM simulation model is developed to jointly analyze the influence of current-induced polarity effects on the evolution of material migration and IMC growth together with experimental results. Specifically, under the influence of the polarity effect, the thickness of the anode IMC layer is approximately twice as thick as that of the cathode. The IMC thickness on both sides is much thicker than the center under the influence of the size effect. Unlike previous studies, the IMC at the commercial bonding interface is mainly the Al_3_Au_8_ phase in columnar crystal morphology and the α-AlAu_4_ phase in nanocrystalline morphology, with the former being mainly located in the middle region of the IMC layer, while the latter is mainly located in the edge region of the IMC layer. Due to the overgrowth of the IMC layer, the tensile mechanical properties of the interface are degraded, and the failure mode transforms from a single neck fracture to a predominant joint detachment. This study complements and improves the research framework of Au/Al interface IMC at commercial chip joints and lays a theoretical foundation for the development of semiconductor chips toward high integration, high density, and high reliability.

## Graphical abstract

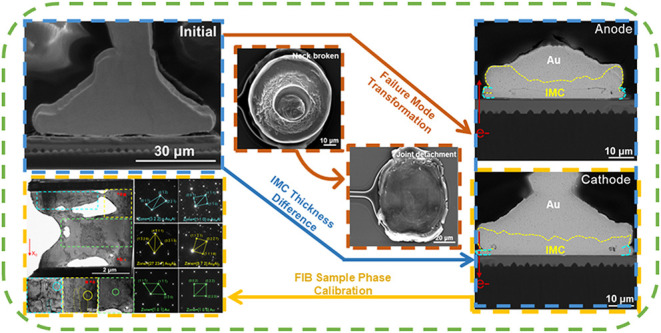

## 1 Introduction

The wire-bonding interconnection is one of the main structures of electronic packaging, playing a vital role in packaging assembly by connecting the internal chip with the external frame to enable electrical connection.^1–3^ Owing to its high process stability, wire-bonding technology accounts for 90% of all packaging processes,^4–6^ predominantly utilizing gold, silver, copper, and aluminum–silicon.^7–12^ Au bonding wires are widely used in cutting-edge integrated circuits because of their excellent ductility, electrical conductivity,^13–15^ and thermal conductivity.^16^ However, with the continuous reduction in the connection size resulting from miniaturized electronic packaging,^17^ the rapid increase in current density can result in severe electromigration (EM) and microstructure variation.^18^ At the same time, the high current density can lead to severe Joule heating effects,^19–21^ dissolution of under-bump metallurgy (UBM), and growth of intermetallic compounds (IMC).^22^ The formation of IMC at the interface is key to a good connection between the wire bump and substrate.^23^ However, owing to the inherent brittleness of IMC, overgrowth can reduce wire bonds and even lead to interfacial failure.^24^ Upon applying an alternating current (±0.6 A) to the Au–Al joints within a dual in-line package, it was found that the electron wind force induced atom movement to the anode, producing compressive stress, reducing the vacancy concentration at the anode and increasing it at the cathode. This stress causes vacancies to migrate from the anode to the cathode, eventually forming voids and cracks.^25^ After bonding the 25 μm diameter Au wire on the Al-1%Si coating of the chip and then applying alternating current (±500 mA) at 170 °C for a 24 h EM test, it was found that under the condition of 1.5 × 10^4^ A/cm^2^ current density at the bond interface, the IMC generated at the interface under the combined thermal-electric action mainly included the Al_3_Au_8_ and AlAu_4_ phase, with a minor presence of the Au_2_Al phase. The overgrowth of IMC can lead to the failure of electronic devices.^26^ The diffusion and formation kinetics of IMC in the solid-phase Al–Au layers were studied by In-situ electron diffraction, revealing that the phase sequence in the solid-state reaction in the Al–Au thin films depended on the initial atomic ratio, with phases successively formed as Al_3_Au_8_→AlAu_4_ when Al: Au = 1:4.^27^ A study of the Au–Al bonding interface microstructures during heat treatment at 175 °C showed that Al_3_Au_8_ was an intermediate product of Al_2_Au and AlAu_4_, becoming the dominant phase before the Al pad was exhausted. After the Al pad is consumed, Al_2_Au is fully converted to Al_3_Au_8_. Subsequently, Al_3_Au_8_ reacts with Au to form AlAu_4_ until AlAu_4_ becomes the only phase between Au and SiO_2_.^28^ In previous studies, the experimental structure was usually designed independently; that is, after the interconnection structure was formed on the basis of pure Al or Al–Si thin films, the interface failure mode and IMC formation process were observed through a high current density or temperature assessment for tens or hundreds of hours. Because there was still a large difference between the structures of traditional chips and commercial chips, it was unknown whether the interface of microscale joints of commercial chips was consistent with this evolution rule.

In addition, as microscale joints connect to electrodes of different polarities for electron input and output during the operation of microelectronic devices, the abnormal growth of the IMCs at both the cathode and anode, namely, the polarity effect, must be considered in the EM testing and evaluation process.^29^ Driven by the electron wind force, metal atoms generally diffuse from the cathode side of the joints to the anode side along the direction of electron motion, resulting in a symmetrical growth of the IMC at the cathode and anode interfaces. Eventually, the open-circuit failure occurs due to the rapid spread of voids and cracks at the interface.^30,31^ The polarity effect has attracted significant attention in studies of the interface between Sn joints and Cu interconnections. The polarity effect on the growth of IMC at the cathode and anode interfaces of Cu/Sn–52In/Cu joints becomes more pronounced with higher temperatures and current densities.^32^ Studies on the polarity effect of EM on the microstructure of the μBGA (ball grid array) joints interface revealed that EM either accelerates or slows down the interfacial reaction kinetics by altering the diffusion of Sn, thus significantly differing the IMC growth rates between the anode and cathode.^33^ By employing the Ni­(P)/Au coating-SnPb joint-Ni­(P)/Au coating interconnection structure, this study analyzed microstructural changes from the perspective of atom directional diffusion under potential difference and chemical potential conditions. It was found that the directional diffusion of Sn along the electron flow, influenced by the EM, caused abnormal IMC growth at the anode interface. While the IMC thickness at the cathode interface remained largely unchanged.^34^ In addition, an anomalous polarity effect was observed at the Sn-9Zn/Cu interconnect interface, where the IMC layer at the cathode was approximately 2.3 μm thicker than that at the anode. This anomaly was attributed to the migration of Sn atoms from the cathode to the anode at higher temperatures, creating a back stress greater than the EM driving force of Zn atoms, which in turn promoted the migration of Zn atoms to the cathode, resulting in the formation of the Cu_5_Zn_8_ and β-CuZn phases.^35^ Current studies on the polarity effect within wire-bonding systems are scarce. However, the EM mechanism at the single Au–Al system interface demonstrated that when the electronic wind force was directed from Au to Al, it hindered the Al consumption rate and delayed failure. Conversely, when directed from Al to Au, it promotes the growth of the IMC phase and accelerates failure. This result showed a trend toward the polarity effects.^36^

A high-throughput DC power supply with a current density of 1.019 × 10^3^ A/cm^2^ was applied to the Au–Al thin layer for about 90 h in the temperature range of 400–500 °C. It was found that regardless of the time, temperature, and current density conditions, only four of the five IMCs may be generated in the Au–Al system, Au_5_Al_2_, Au_2_Al, AuAl, and AuAl_2_. However, the product layers were dominated by the first two.^37^ Driven by the increasing miniaturization of devices, the polarity effect of EM has become a key issue in chip packaging. However, the polarity effect of the EM, the interface structure, and the failure mechanism of the bonds in the actual service of the Au–Al system remain unclear.

Commercial chips typically feature a coating on the Al layer, which serves as an Al migration barrier to inhibit the rapid growth of the Au–Al IMC. Therefore, in order to explore the EM polarity effect of commercial chip-end Au/Al bonding wires in a long-time high-density current service environment, in this study, the growth evolution mechanism of the IMC layer at the commercial chip-end Au/Al interface is dissected. The microstructural evolution and defect formation of different polarity joints before and after EM are characterized and analyzed, and the degradation mechanism of mechanical properties and the transformation of failure modes under the effect of EM are investigated.

## 2 Experimental Procedures

In this experiment, a 4N–Au wire with a diameter of 20 μm was used to bond commercial Sanan S-118BBMUD light-emitting diode (LED) chips, as shown in Figure S1a. The original chip dimensions are shown in Figure S1b. The rated current of the chip was 60 mA, the rated power was 0.2 W, the rated voltage was 3.0–3.2 V, and the wavelength of the blue lamp was 450–452.5 nm. The cross-sectional diagram of the bonded chip is shown in Figure S1c. A visible metal layer under the bond is shown in Figure S1d. Given the rated current requirements of the chip, the DC current used in the EM test was 60 mA. Upon energizing, the current densities crossing the bonding Au wire and Au/Al interface were 1.96 × 104 A/cm^2^ and 0.31 × 104 A/cm^2^, respectively, and the current density at the bonding wire was higher. As shown in Figure S1a, the Au wires connect the two electrodes (P and N electrodes) on the chip side to the substrate through ultrasonic bonding, forming a current path. The direction of electron movement in the current circuit is shown in Figure S1a. On the chip side, the joint at the P+ pole is the anode (2#), while that at the N- pole is the cathode (3#). The two wedge-shaped bonds on the substrate side are not described in this study. Therefore, the bonds referred to herein are ball bonds (2# and 3#) on the chip side.

To observe the interfacial IMC before and after EM, the samples were polished directly. Subsequently, the diffusion distances of the migrating elements and IMC thickness variations, as well as the microstructures and elemental proportions of the microjoint interface, were analyzed using a scanning electron microscope (SEM) and energy-dispersive spectrometer (EDS). To further clarify the structure of the IMC, the interface was sectioned by using a focused ion beam (FIB-Ga ion source). Figure S2a–d shows the location and process of the FIB sample cutting. After the sample preparation, the interface was characterized by electron backscatter diffraction (EBSD) and transmission Kikuchi diffraction (TKD) using the FEI Versa 3D scanning electron microscope. At the same time, the phase structures were analyzed using the FEI Tecnai G2 F30 transmission electron microscope via brightfield (TEM-BF) and selective area electron diffraction (SAED).

The bonding strength tester (PTR-1102) was used to perform tensile tests on the interconnect structures postbonding at a tensile speed of 0.1 mm/s to evaluate the change in bond strength at the interface of the joints after different durations of EM testing. Each sample was tested at least 6 times to ensure the reliability of the data. During the tests, the displacement force curves were recorded for further adhesive mechanical properties and fracture energy analysis, and the area was obtained from the integration curves.

Furthermore, ANSYS Workbench 2021-R1 finite element analysis (FEA) software was used to simulate the current density under EM conditions. In order to reduce the computational load, the cross-section and material distribution of layers in the 3D model were simplified, as shown in Figure S1c,d. For simplicity, the wire diameter of the Au was 20 μm, the diameter of the first bonded ball was about 60 μm, and the metal layers under the bond were two layers of Ti/Ni. 0.1 μm of the IMC layer is placed on the Au–Al interface bonded on the chip side, as a very thin IMC layer will be generated during the bonding process. To simulate the real EM test conditions, the FEA calculations were performed by using the thermal-electric module of ANSYS, with an anode current of 6 × 10^–2^ A, a cathode voltage of 0 V, and a convection boundary condition with an overall model surface temperature of 22 °C. The heat generation inside the chip was 26 W/μm^3^.

## 3 Results and Discussion

### 3.1 Initial Bonding Interface Characterization

The bond composition at the chip side and the microstructure of the metal layers under the bonds were analyzed in their initial states (Figure 1a–c). According to the results of secondary electron images and EDS mapping, the different layers on the chip side are mainly composed of Al, Ti, Ni, and Ga (representing GaN). Here, Ti and Ni served as bonding and diffusion barriers, respectively, while the Al-rich layer adjacent to the Au bump formed a post bond. Because the cathode and anode on one side of the chip use the same process, their initial structure is identical to that of their respective joints before electromagnetism. As shown in Figure 1c, there is a thin IMC layer with a thickness of only 200 nm between the Ti layer and the Au bump. EDS point scanning of the IMC showed that the main components were Au and Al. At P1, the atomic ratio of Al:Au was about 1:4, whereas at P2, the atomic ratio is about 3:8. Combined with the phase diagram of the Au–Al alloy shown in Figure S3,^38^ the IMC phase at P1 is most likely the AlAu_4_ phase, while the phase at P2 is most likely the Al_3_Au_8_ phase.

**Figure 1:**
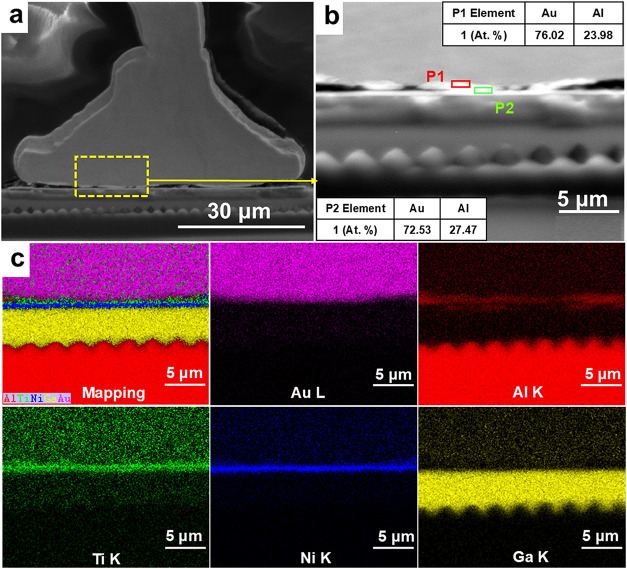
Initial interface morphology and surface scanning results of the Au wire bonded joint: (a) microstructure, (b) the structure of interfacial element layers and spot scanning results of UBM and Au joint, and (c) EDS mapping analysis results of the yellow dotted box.

### 3.2 Interfacial Microstructures after EM

#### 3.2.1 Microstructure of Interfacial IMCs

Figure 2a–f shows the interface microstructural morphologies and elemental distribution of the cathode and anode joints after 1000 h of current stressing, as well as the elemental line scanning results. It can be seen that the thickness of IMC at both the anode and the cathode centers is thicker than that at the edges. Further magnification of the microstructure at the interface and line scanning analysis across the interface are shown in Figure 2b,c,e,f. According to the EDS-line test results, the average thickness of the anode IMCs is 5.7 μm, while that of the cathode IMCs is 5.5 μm, and the thickness of the cathode is slightly lower than that of the anode. The EM process caused local current crowding at the Au–Al interface, leading to a local temperature increase and increased elemental migration.^39^ In Figure 2b,e, the IMC layers at the anode and cathode show different morphologies. Small voids and intermittent short cracks are present at the anode interface, whereas no voids and cracks are observed at the cathode. The results of defect structure formation are also different from the two poles.

**Figure 2:**
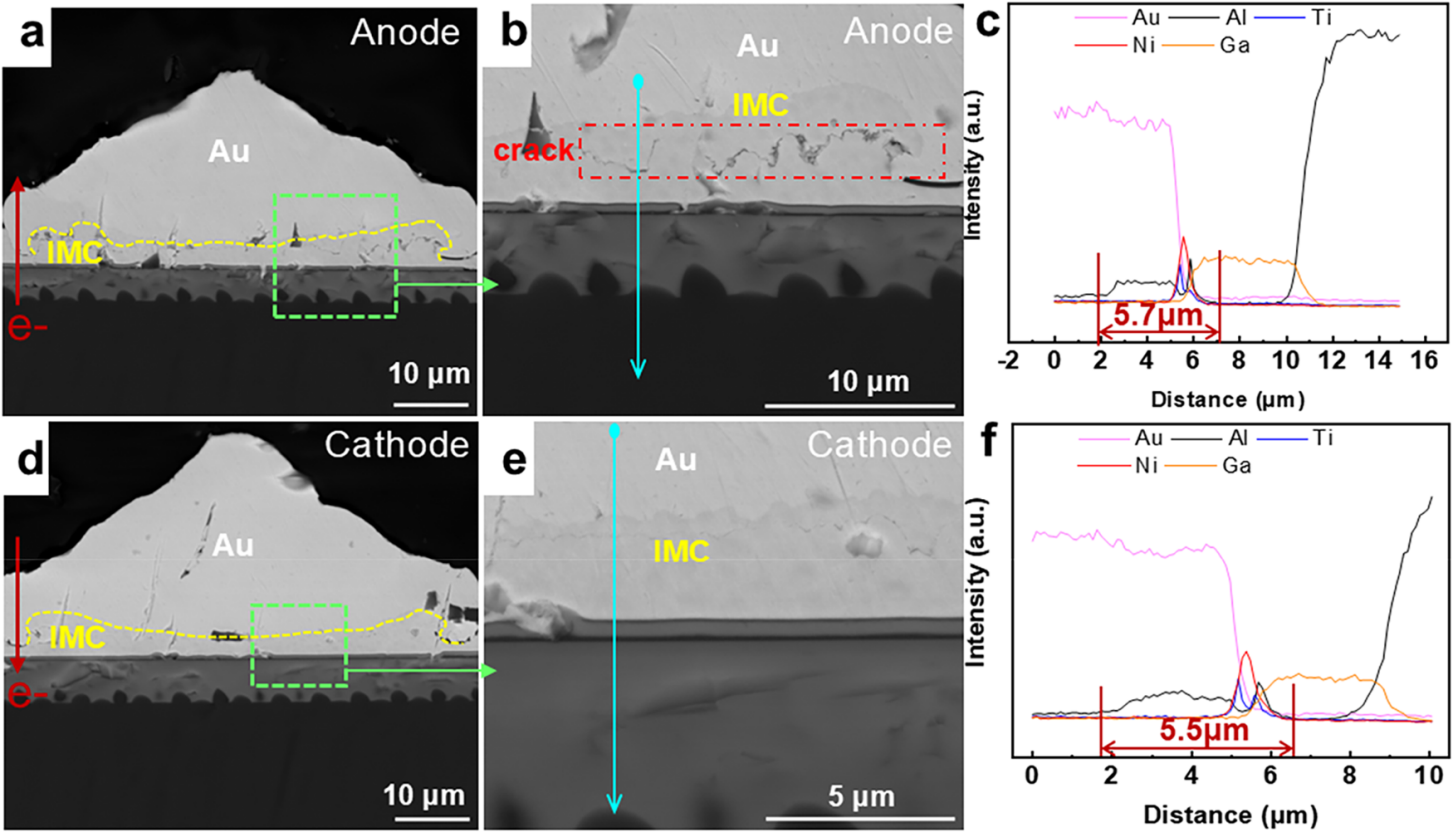
Morphology and element distribution of the chip-side bonds after 1000 h of EM: microstructure morphologies of (a, b) the Au anode and (d, e) the Au cathode and line scanning results at the blue position of (c) the anode and (f) the cathode interfaces.

When the current stressing time was extended to 2000 h, the results are shown in Figure 3. Figure 3a–h shows that the IMCs on the Au–Al interfaces of the cathode and anode increased significantly after 2000 h of EM. In addition, the difference in IMC thickness between the center and the edges also increases significantly. According to the EDS-line test results, the average thickness of the anode IMC is 8.7 μm, while the average thickness of the cathode IMC is 6.3 μm (Figure 3d,h). The thickness of the anode IMC is significantly thicker than that of the cathode IMC. The anode shows a strong capacity for Al atom migration and diffusion. Figure 3b,c,f,g clearly shows that the cathode and anode IMC layers exhibit different microstructural morphologies. In the anode IMC layer, voids and internal continuous cracks are expanding, while cracks and micropores begin to appear on both sides of the cathode IMC layer. The formation of cracks and voids is attributed to the combined effects of the electron wind force, electrochemical potential gradient, and temperature gradient. This combined effect had different impacts on the cathode and anode sides, promoting the migration of Al atoms from the interface to the joint interior. However, the different diffusion rates resulted in a thicker IMC layer at the anode interface than at the cathode. The growth of interfacial compounds shows a polarity effect.

**Figure 3:**
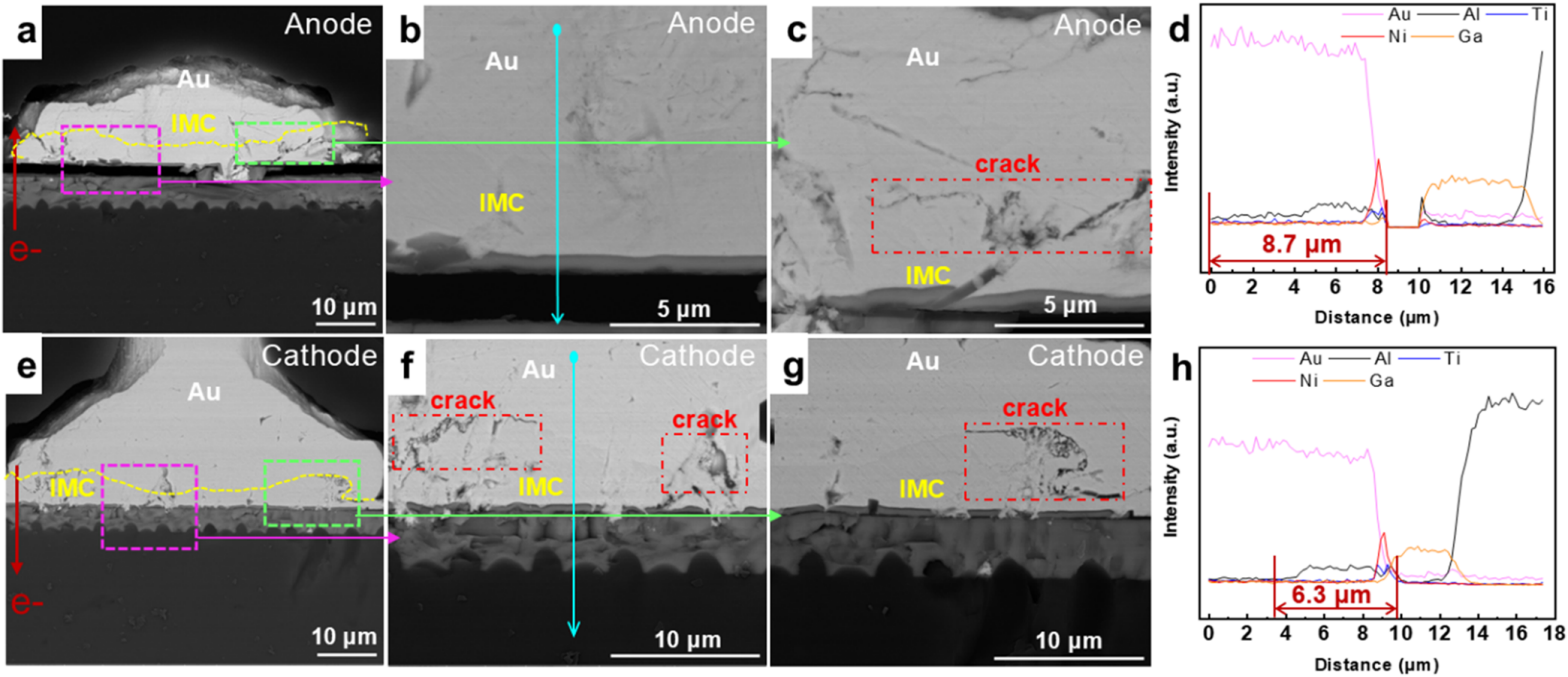
Morphology and element distribution of the first joint after 2000 h of EM: microstructure morphology of (a–c) the Au anode joint and the UBM, microstructure morphology of (e–g); the Au cathode joint and the UBM, and line scanning results at the blue position of (d) the anode and (h) the cathode interfaces.

The microstructural morphology and element distribution of the bonding interface can be seen after extending the current action time to 3000 h, as shown in Figure 4a–f. Figure 4a,b shows a clear, continuous, uniform crack through the anode. Figure 4d,e shows a small crack in the middle of the cathode, accompanied by distinct bending cracks on both sides. As shown in Figure 4a,d, due to the volume expansion caused by IMC overgrowth and phase transformation, the bottom of the bonded joint protrudes beyond its original shape. As can be seen in Figure 4c,f, the diffusion distance at the anode has sharply increased compared to that at 2000 h of EM and is significantly larger than that at the cathode. The diffusion distance of aluminum at the anode interface is about 11.2 μm, while at the cathode interface, it is about 6.6 μm.

**Figure 4:**
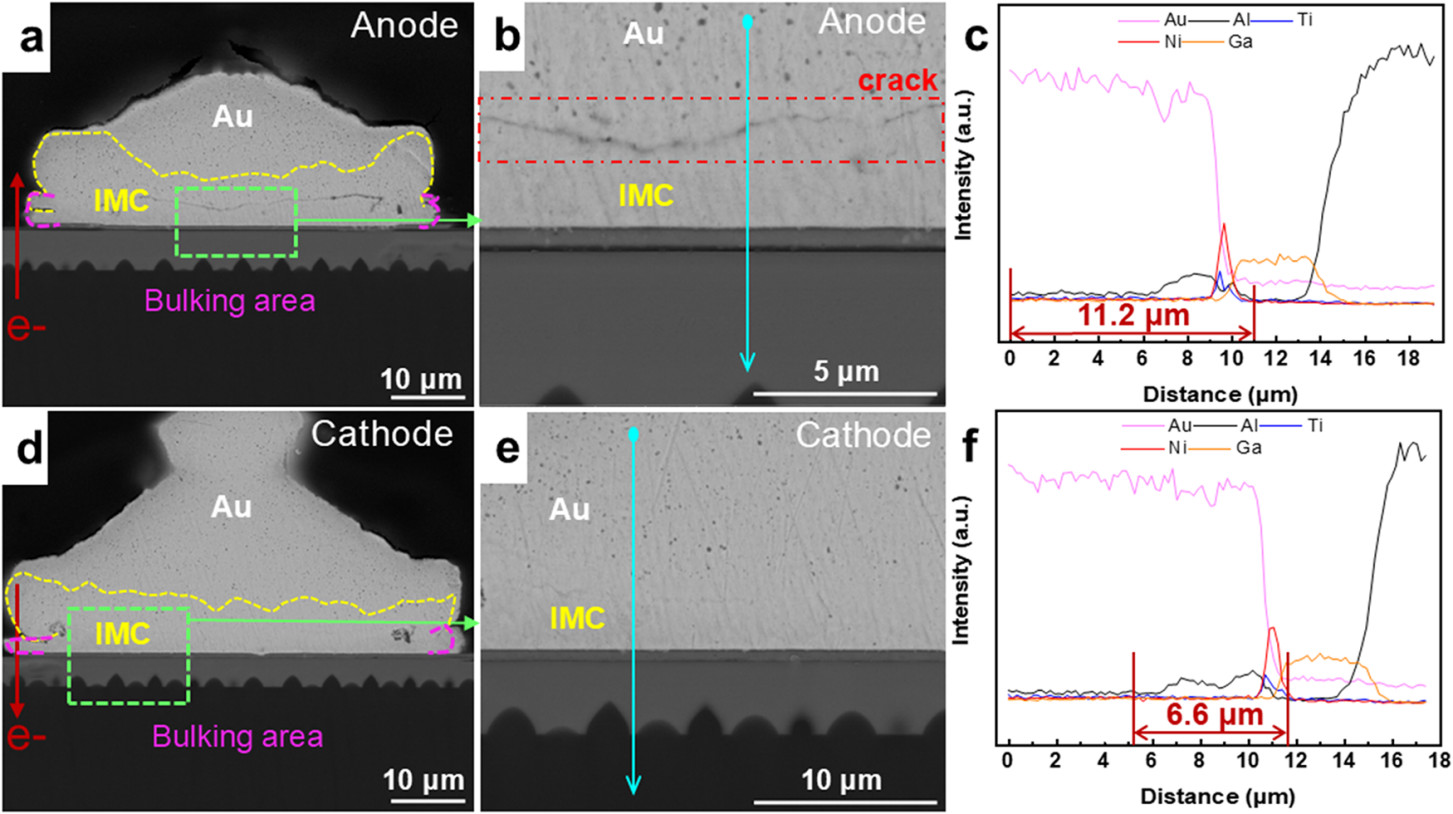
Morphology and element distribution of the chip-side bonds after 3000 h of EM: microstructure morphologies of (a, b) the Au anode and (d, e) the Au cathode and line scanning results at the blue position of (c) the anode and (f) the cathode interfaces.

#### 3.2.2 Interfacial IMC Growth

Figure S4 compares Al diffusion distances, which represent the IMC growth, and the growth rates at the cathode and the anode. After 1000 h of EM, the IMC growth rate at the cathode was about 0.50 μm/100 h, while the IMC growth rate of the anode was about 0.52 μm/100 h. The IMC of the anode was slightly thicker than that of the cathode and grew correspondingly faster. After 2000 h of EM, the IMC growth rate of the cathode decreased to approximately 0.08 μm/100 h, while the IMC growth rate of the anode remained higher at about 0.30 μm/100 h. This marked a significant decrease in the growth rate, indicating that the IMC growth rate is significantly reduced at both the anode and cathode, but more so at the cathode. After the current stressing time was extended to 3000 h, the IMC growth rate of the cathode was about 0.03 μm/100 h, and the IMC growth rate of the anode was about 0.25 μm/100 h. The IMC growth rate keeps decreasing as the IMC thickness increases. The overall IMC shows a power–law relationship with the increase of time. The difference in the effect of electron wind force in the two polar joints from the initial stage of bonding to the 1000 h process of EM testing (anode IMC layer thickness ranging from 200 nm to 5.7 μm and cathode IMC layer thickness ranging from 200 nm to 5.5 μm) is not significant, with the force caused by the temperature gradient and chemical potential gradient as the main driving force. And there is no difference in the action of this main driving force in the two polar joints. Therefore, the cathode and anode IMC layer growth rates are similar in this stage. In the subsequent stage, with the increase of EM test time, the electron wind force gradually becomes dominant. And there is a significant difference in the effect of electron wind force in different polarity joints, that is, the growth of the anode IMC layer is promoted, while the growth of the cathode IMC layer is inhibited. As a result, the anode IMC layer growth rate was significantly higher than the cathode IMC layer growth (22% higher).

#### 3.2.3 IMC Crystal Structure after Current Stressing for 1000 h

In order to investigate the effect of electromagnetism on the growth and evolution of the IMC phase, TEM and TKD analyses of the FIB samples at the cathode interface were carried out, and the physical phase results are shown in Figure 5. Figure 5a shows the TEM crystal morphology of the IMC located between the Au layer and the UBM layer. Figure 5e,f presents the SAED results of the nanocrystalline α-AlAu_4_ phase located in the blue dashed box depicted in Figure 5a. Figure 5h,i shows the SAED results of the columnar grain Al_3_Au_8_ phase located in the yellow dashed box in Figure 5a. Figure 5k,l shows the SAED results of the Au phase located in the green dotted box in Figure 5a. Figure 5g,j,m depicts the crystal structures of the AlAu_4_ phase, Al_3_Au_8_ phase, and Au phase. Figure S5a shows the results of the distribution region of each phase in the IMC layer of the whole FIB sample. Figure S5b provides an additional explanation for the TKD results of Figure S5a.

**Figure 5:**
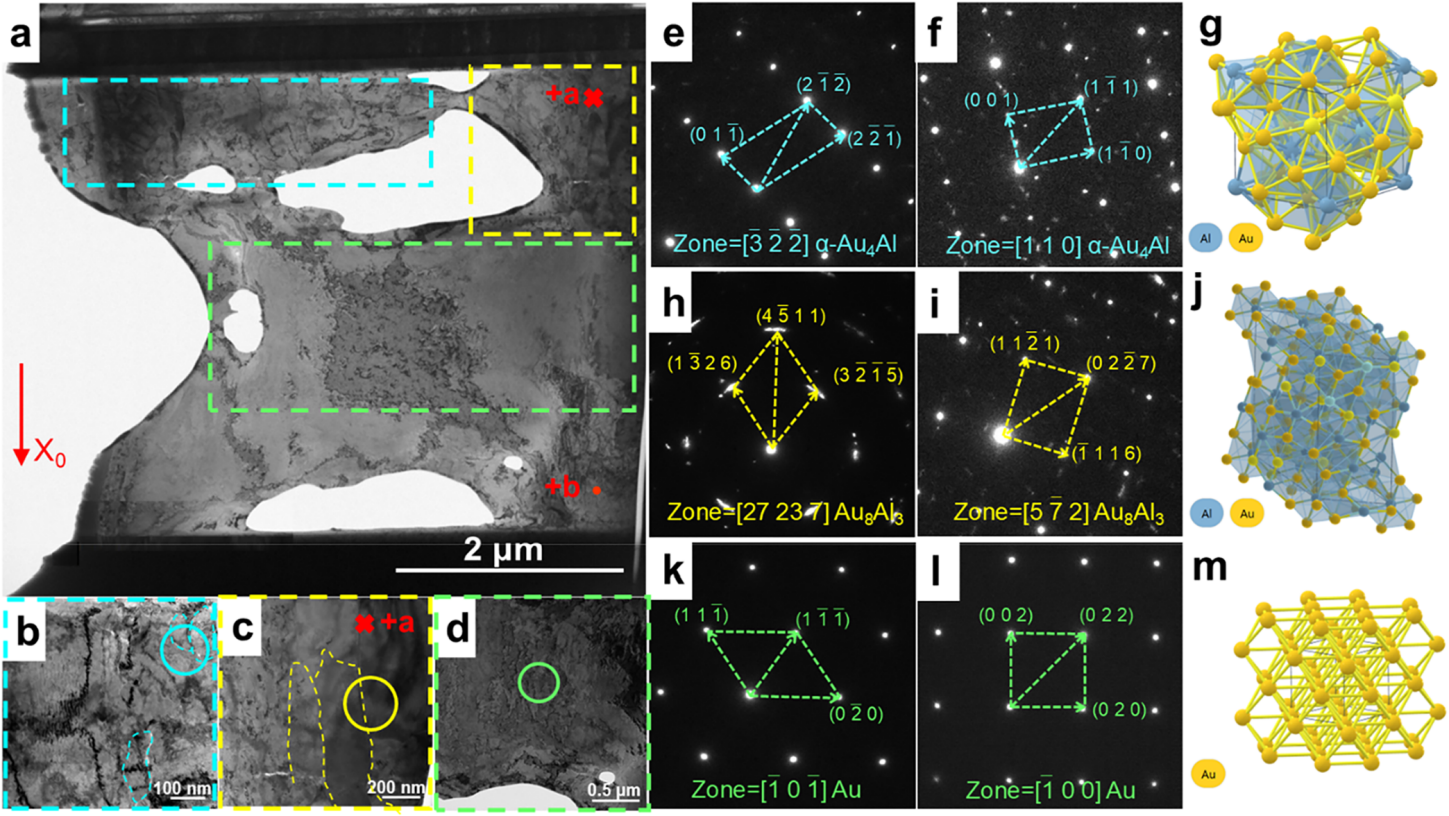
TEM results of cathodic interface FIB samples: microstructure morphology of (a) overall FIB sample (b) nanocrystalline α-AlAu_4_ on both sides, (c) columnar grain Al_3_Au_8_ in the middle region and (d) Au matrix, SAED results of (e, f) the nanocrystalline α-AlAu_4_ phase, (h, i) columnar grain Al_3_Au_8_ phase, and (k, l) Au phase and crystal structures of (g) the nanocrystalline α-AlAu_4_ phase, (j) columnar grain Al_3_Au_8_ phase, and (m) Au phase.

Based on the above characterization results, the nanocrystalline region enclosed in the blue box in Figure 5 was identified and verified as AlAu_4_. Its crystal parameter is 6.903 Å, which is similar to the α-AlAu_4_ lattice. Diffraction analysis shows that the columnar crystal region in the yellow box corresponds to Al_3_Au_8_ with lattice parameters *a* = 7.725 Å, *c* = 41.717 Å, α = β = 90°, and γ = 120°. Based on the IMC morphology analysis shown in Figures 2–4, the phases on both sides of the bond are identified as α-AlAu_4_ nanocrystals, whereas the central region consists of Al_3_Au_8_ columnar grains. Table 1 lists the crystallographic parameters of each phase obtained from the Materials Project (MP) and calibrated with TEM data. From the table, it can be seen that in the center region at the interface position, there is a significant lattice volume difference between the Al_3_Au_8_ new phase and the Au matrix, with a volume expansion as high as 36.22. Consequently, a large volumetric strain is generated during the growth process, which results in the formation of interfacial microcracks. During the formation of the α-AlAu_4_ phase on both sides of the joint, a significant difference in lattice volume between the α-AlAu_4_ phase and the Au matrix results in an expansion rate of 4.85. The expansion of IMC unit volumes on both sides leads to material accumulation and enrichment, promoting outward and lateral growth, which eventually leads to pronounced cracks on both sides. This outward and lateral growth contributes to significant cracks. This clarifies the fundamental cause of IMC edge cracking observed in Figures 2–4.

**Table 1:** Lattice Parameters of the IMCs at the Au–Al Interface

type	Au	Al	Al_3_Au_8_	α-AlAu_4_
crystal system	cubic	cubic	trigonal	cubic
lattice by MP	*a* = 4.17 Å	*V* = 65.89 Å^3^	*a* = 7.84 Å, *c* = 42.89 Å	*a* = 6.98 Å
	*V* = 72.58 Å^3^		*V* = 2285.39 Å^3^	*V* = 339.82 Å^3^
lattice by TEM	*a* = 4.078 Å		*a* = 7.725 Å, *c* = 41.717 Å	*a* = 6.903 Å
	*V* = 67.8 Å^3^		*V* = 2155.96 Å^3^	*V* = 328.93 Å^3^

Figure S6a,b shows the results of grain orientation analysis for each region of the chip-side bond after EM. Based on TEM diffraction and IMC phase distribution results, the +a position represents the columnar grain Al_3_Au_8_ phase located in the bonding center. The predominant grain orientation in this region is (011̅0), with a texture intensity of 21.37. On both sides of the bond, there is a nanocrystalline α-AlAu_4_ phase with a random grain orientation along the *X*_0_ *Y*_0_ plane. Nevertheless, according to pole figure analysis, it shows a preferred orientation of {111}∥*Z*_0_. The + b position corresponds to the columnar grain of the Au matrix and the grains show (001) < 001> orientation. There is the {001}∥*X*_0_ preferred orientation with a texture strength of 47.55.

### 3.3 Au Bond Grain Evolution under Current Stressing for 0–3000 h

EBSD tests were performed on the first joints at different EM stages to analyze the structural evolution throughout the EM process. Grain morphologies and orientations are depicted in Figure 6a–h. In Figure 6, the grains at the edges of both the cathode and anode joints are fine equiaxed grains. The grains at the microscale joints were significantly enlarged after 1000 h of EM, which led to the deterioration of the mechanical properties and reduction of the interfacial bond strength. As the EM process continued, the size and area proportion of columnar grains in the Au matrix core increased, and the grain orientation changed from random to <001> orientation. The tendency to preferential orientation increased as the EM process proceeded, which could offset the degradation of properties caused by some of the IMC phases. This helps to maintain the plastic strength of the bond in the longitudinal *X*_0_ direction. After 2000 h of EM, the grain size and orientation in the core region remained unchanged, while the proportion of grains in the edge region increased slightly. The difference in the grain structure between the core and edge regions may lead to stress concentrations, which could degrade the mechanical properties of the edge region. Zhang et al.^40,41^ investigated the diffusion and solidification of CuGB/Al solid–liquid systems with varying Cu phase angles and identified the characteristics of grain inheritance. The structure and properties of the interface are highly dependent on the orientation of the underlying solids. Based on the TEM results, it was observed that the core of the Au bonds and the IMC near the core had a columnar grain structure, while the IMC near the edge and the edge region had a nanocrystalline grain structure. It is speculated that during the material migration process of EM, the entry of Al into the Au matrix was influenced by the original grain structure on the bottom surface of the Au joints. Al entering the nanocrystalline grains was more likely to form α-AlAu_4_, while the columnar crystal structure tended to form Al_3_Au_8_. During the element diffusion process at the interface, the grain morphologies of the IMC and the original structures of the joints remained highly consistent along the longitudinal *X*_0_ direction, showing obvious characteristics of grain inheritance despite the phase transformations and volume expansion that occurred.

**Figure 6:**
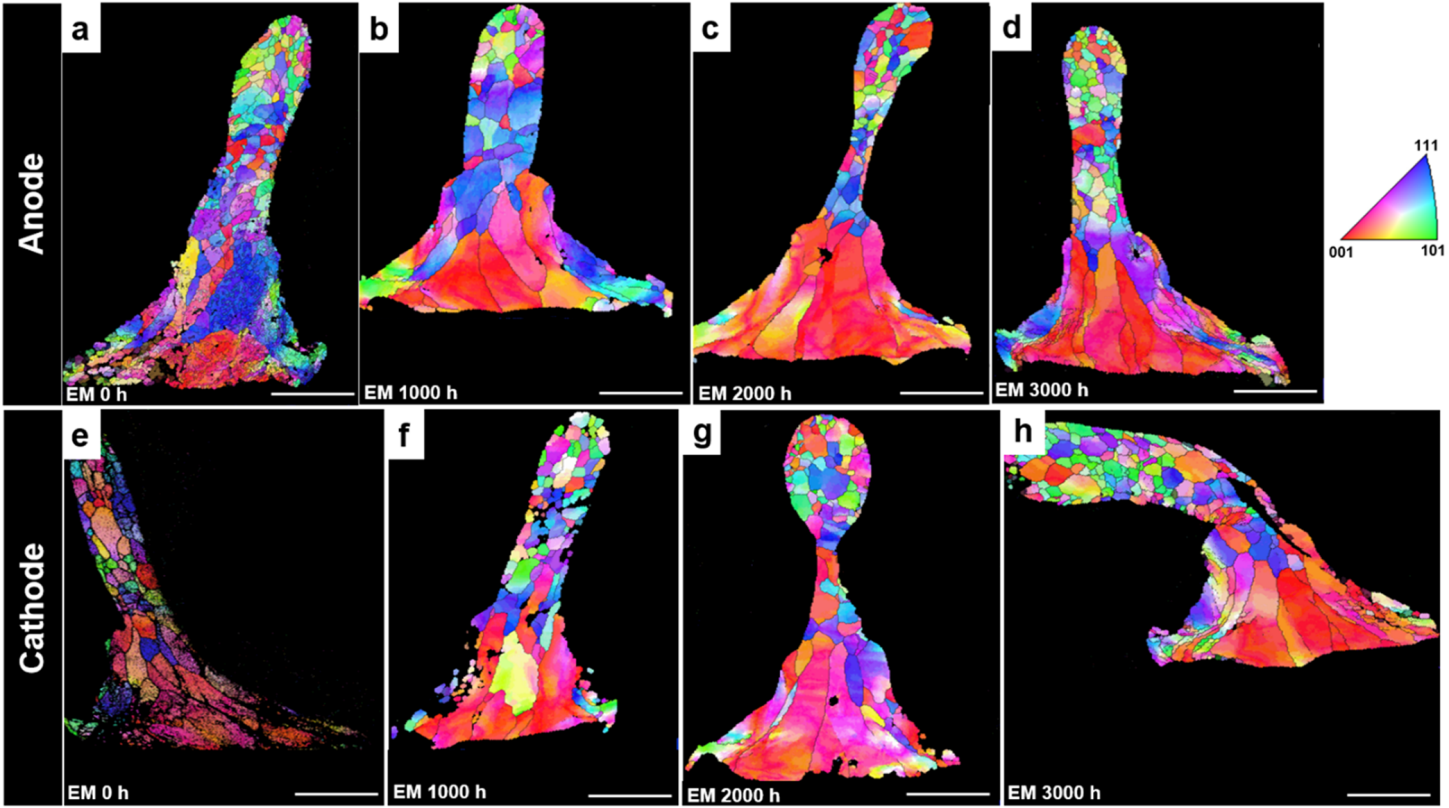
Evolution of the overall structure of the microscale joint under different EM stages: At the same scale bar of 20 μm, the structures of (a–d) the anode of the Au microscale joint and (e–h) the cathode of the Au microscale joint.

To further analyze the internal stress distribution within the joint, the IMC layer of the first joint post-EM was analyzed for Kernel Average Misorientation (KAM), as shown in Figure S7. High defect densities were mainly observed in the columnar grain Al_3_Au_8_ region in the middle of the joint (outlined in blue) and the nanocrystalline α-AlAu_4_ region on both sides of the joint (outlined in yellow). Additionally, the average defect density in the nanocrystalline α-AlAu_4_ region in the middle of the joint is significantly higher than that in the columnar grain Al_3_Au_8_ regions on both sides. The level of internal stress is much lower in the Au side regions. This internal stress contributes to lower tensile and yield strength as well as increased brittleness. Consequently, the region around the black hole is highly susceptible to initial failure and separation, which are attributed to microscopic defects caused by the large number of internal stresses generated during the FIB sample preparation process.

### 3.4 Mechanical Property Deterioration

#### 3.4.1 Bonding Strength and Fracture

The tensile tests were conducted on the bonded joint at a speed of 0.01 mm/s to observe the mechanical deterioration after EM. Figure 7a–g shows the maximum tensile force and fracture energies (energy required to break the Au wire bond) of the Au wire bonds at the anode across different EM stages. Figure 7a shows that the average tensile force was 6.58 gf. After EM exposure, the value slightly decreased to 6.31 gf at 1000 h and to 4.60 gf at 3000 h. The tensile force first increases and then decreases subsequently with the increase of EM time, which is similar to the fracture energy in Figure 7b. The results show that the bond strength after bonding does not achieve the optimal value. However, the mechanical properties peaked after 1000 h of energized use and then deteriorated with the increase in energization time. The corresponding fracture energy data (integral force–displacement curve) exhibit a similar trend. Combined with the interfacial IMC evolution during EM discussed above, the initial average IMC thickness at the anode was 0.5 μm, which increased to 11.2 μm after 3000 h of EM. After 1000 h of EM, the IMC thickness reached 5.7 μm, which strengthened the connection between the Au wire and the pad and slightly enhanced the mechanical properties of the joint. However, with the increasing IMC thickness, microholes and cracks appearing in the layer significantly reduced the mechanical properties of the joint. With the thickening of the IMC layer during the EM process, the Kirkendall effect intensified, leading to the enlargement of the holes and cracks. Additionally, the formation of new phases, α-AlAu_4_ and Al_3_Au_8_, in different regions of the joint resulted in stress concentrations on both sides, which reduces the interfacial bonding capability.

**Figure 7:**
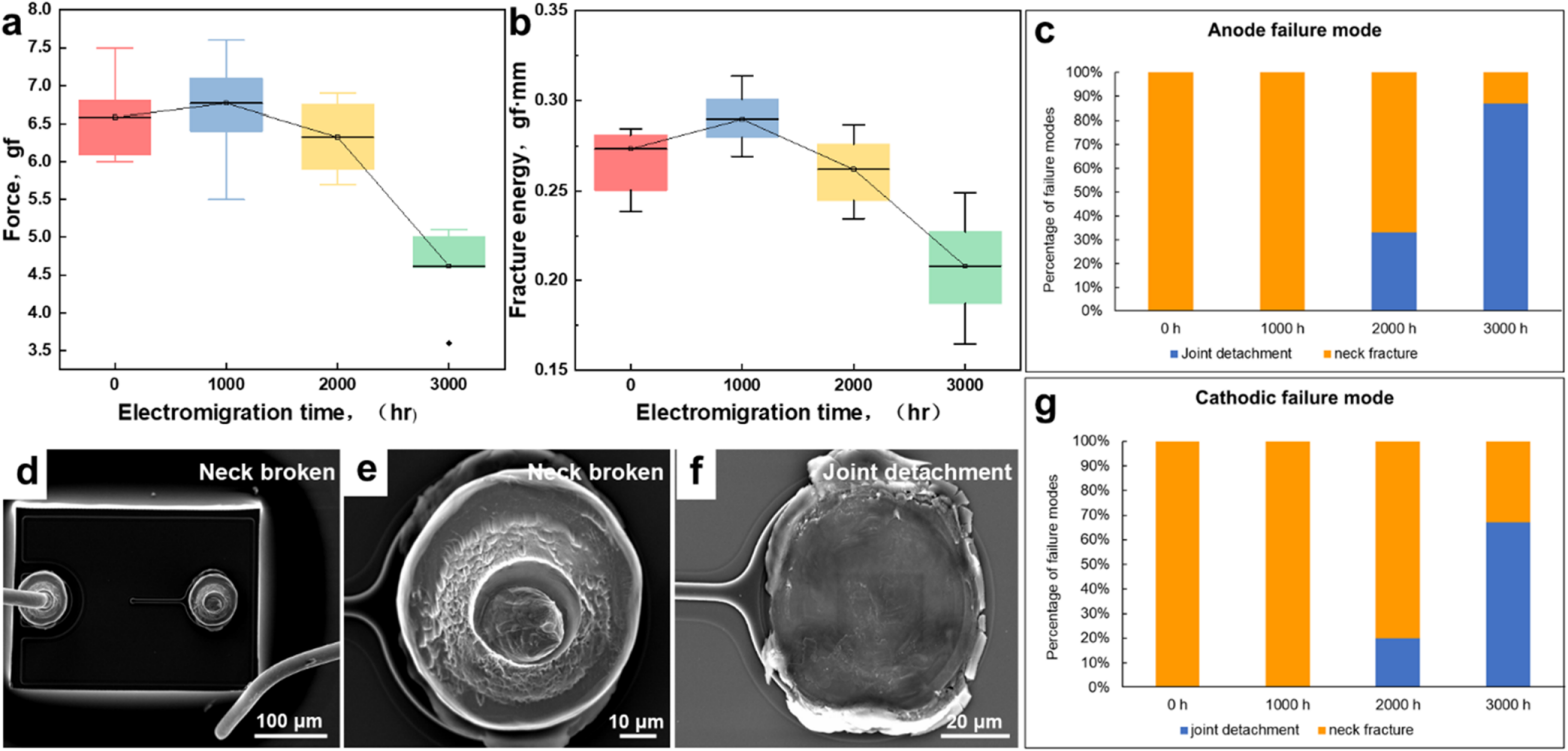
Tensile force of gold wire bonds at different stages: (a) tensile force, (b) tensile fracture energy, percentage of failure modes at (c) anode and (g) cathode, and (d–f) fracture surfaces showing different failure modes.

Accordingly, the fracture location of the bonding structure also changes as the EM progresses. The fracture location transfers from the neck of the bonding wire to the detachment of the bonding joint at the chip side (see Figure 7d–f). These two cases are termed wire neck fracture and joint detachment, respectively. Due to the electromigration behavior, the bonding joint becomes an anode and cathode. Therefore, the percentage of failure modes in which failure occurs at the anode and the percentage of failure modes in which failure occurs at the cathode are shown in Figure 7c,g.

The initial failure modes of both cathode and anode, as well as the failure mode after 1000 h, were single neck fractures. This is due to the optimal thickness of the IMC layer at the bonding interface, which optimizes bonding strength and mechanical properties and ultimately leads to neck fracture because the neck has the smallest cross-sectional area. After 2000 h, both the cathode and anode show a new failure mode: the detachment of the chip-side bond, namely, joint detachment. This is attributed to microcracks in the IMC layer at the interface during the growth process, which gradually formed a crack source. After 3000 h, detachment at the chip side became the primary failure mode for both the cathode and anode. This is due to the further overgrowth of the IMC layer and the lateral compression of AlAu_4_ IMC at the edge that exacerbated the interfacial stress concentration. The formation of hard and brittle IMC phases leads to penetrating internal cracks that rapidly reduce the matrix’s bonding strength. Consequently, the fracture mode transforms into an interfacial fracture. After 3000 h of EM exposure, the differing driving forces at the cathode and anode result in more extensive lateral growth of IMC at the anode edge than at the cathode, increasing the likelihood of joint detachment on the anode side.

### 3.5 Crystal Whisker and Vacancy

The fractures, both before EM and after 1000 h of EM, are neck fractures, yet their fracture mechanical properties exhibit significant differences (Figure S8a–i). There is some Al extruded from the pad at the edge of the fracture prior to EM (Figure S8a). Traces of plastic deformation caused by the fixture compression during the joint formation process are visible on the side (Figure S8b). The macroscopic fracture surface exhibits distinct signs of necking and plastic deformation. As illustrated in Figure S8c, the traces of slip lines are visible on the magnified fracture surface in Figure S8a. After 1000 h of EM, metal flocs can be seen between Au and pad Al, regardless of whether it is the cathode or the anode (Figure S8d,g). Further enlarging these flocs, the result is as shown in Figure S8e, which look like rods growing between Au and pad Al, knotting at the tops. Elemental composition analysis at P1 in Figure S8e indicates that it primarily consists of Au and Al, along with minor amounts of Ti and Ga. In light of the preceding conclusion, substantial compressive stress is evident at the bonding interface. These rod-shaped metal protrusions, formed by the migration of Au and Al atoms under compressive stress, are likely whiskers. Based on the morphology, it is deduced that the gold and aluminum originate from the pad and gold wire, respectively. The gold wire experiences significant compressive stress during the formation of Au bumps and compound transformation, providing a metal source and pressure for the growth of whiskers. Consequently, whiskers can rapidly grow under the combined effects of thermal stress, compressive stress, and concentration gradient.

Further enlargement of the center of the fracture surface in Figure S8d, as shown in Figure S8f, and a large number of exposed voids can be seen. Specifically, large voids and clusters of microvoids are identified at the cathode fracture. Meanwhile, granular phases appear on the central fracture surface of the anode (see Figure S8h,i). Compared with the smooth fracture surface before EM, the granular surface is more prominent. This is very similar to the EM phenomenon found in Sn-based solder interconnection, where compounds accumulate at the anode and form voids at the cathode.

### 3.6 Mass Migration

#### 3.6.1 Al Atom Migration and Edge Effect

From the EM results presented above, Al and Au are the main migration elements within the Au–Al bonds. Given that Au is the matrix metal, diffusion of Al inevitably occurs in Au. Therefore, by observing Al, we can infer the behavior of Al in reverse. Thus, Al is the element to which we pay attention to. Among the Au bonded LED chip, metal Ti and Ni as the diffusion layer are quite stable during the current stressing process. Al atom may diffuse to the Ti\Ni layer at the beginning of EM. Once the atomic concentration reaches saturation and cannot break through the barrier layer, migration will inevitably occur in a direction away from the interface, leading to a diffusion steady state. This diffusion will make the Al–Au IMC continuous growth until it reaches a steady state for diffusion.

When an electric current is applied to the bond, the Al mainly here, within the Au bond, is subjected to the electron wind force *F*_em_ definitely bumped by the directional moving electrons. Al atoms may migrate driven by this force. At the same time, the Al atom migration process is usually also affected by the force caused by temperature (T) gradient, back stress (σ) gradient, and chemical potential (μ) gradient simultaneously. Write them as *F*_T_, *F*_σ_, and *F*_μ_, respectively, as shown in eq S1.

Temperature difference, stress difference, and the chemical potential difference will finally reflect on the chemical concentration difference in atom flux flowing through the cross-section of the bonds. So, it is reasonable to simplify the above formula using atom flowing flux to be eq S2.

Assuming the migration process reaches a steady state after 1000 h, the Al migration direction is always away from the chip side, along the bonding wire, toward the substrate end. This is true in both the anode and cathode, deduced from the line scanning results from section 3.1.

Taking the 2# anode bond as an example, at the IMC/Au interface, driven by the chemical concentration difference, upon power-on, the Au atom initially diffuses toward the chip side along the red arrow in Figure S9a. However, as the power-on time increases, Au atoms accumulate in the diffusion barrier layer, eventually reaching saturation. As migration persists, the concentration of Au atoms surpasses the saturation level and then moves in the direction of the black arrow away from the chip, driven by the chemical potential gradient (concentration gradient and temperature difference at the same time).

#### 3.6.2 Electromigration

When examining the effect of current stressing, we focus on the migration direction and magnitude of the migration force of Al and Au atoms driven by electronic wind. The force *F*_em_ acting on a diffusing atom is taken to be eq S3.^42^ The effective charge number Z* can be further written as eq S4. Conceptually, it means that to calculate Z*, we need to know the specific resistivity ratio of a diffusing atom to a lattice atom. Typically, for metallic conductors such as Al and Cu, their Z* is about 1 order of magnitude larger than the number of valence electrons of the metal atom. Physically, we expect so because the cross-section of collision of a diffusing atom at the activated state is about 10 times larger than that of an atom in the equilibrium state. That is, eq S4 can be further simplified to eq S5. When diffusion, no matter what forces drive it, occurs under constant temperature conditions, γ represents a numerical value associated with diffusion driven by the concentration gradient at a specific temperature. At the onset of electromigration, it can be assumed that the concentration gradients of Al atoms, Au atoms, and the surrounding medium are all at their maximum values, tending toward consistency.

When diffusion occurs at a constant temperature, γ is a value related to the diffusion driven by a concentration gradient at a certain temperature. At the beginning of electromigration, it can be assumed that the concentration gradients of Al atoms, Au atoms, and the surrounding medium are all at their maximum values and tend to be consistent. The Z* of Al (*Z*_Al_*, assuming Δ*H*_m_ = 0.62 eV/atom^42^) is approximately −58.2. The diffusion activation energy of Au ranges from 0.2 to 0.5 eV, and its calculated *Z*_Au_* falls between −16.7 and −46.3 (**Calculation Details** in the Supporting Information). In terms of numerical comparison, Al exhibits a higher effective charge than Au at the same temperature.

Further, by substituting *Z*_Al_* and resistivity ρ_Al_ at the migration temperature into eq S3, we obtain the ratio of electron wind forces acting on atoms Al and Au under electrified conditions, which is between 4.5 and 12.3, *F*_EM_^Al^/*F*_EM_^Au^ = 4.9–13.4. That is, the driving force acting on the Al atom is at least 4.9 times that of the Au atom induced by current stressing in this test.

This means that Al atoms will move along the direction of electron flow due to the electron wind, while Au atoms will find it challenging to move. The vacancies left by the migration of Al atoms generate back stress, which makes it even harder for Au atoms to move under the influence of the electron wind. Initially, Al atoms did not experience this force. Once Al atoms gain the initiative and move under the electron wind, the conjugated Au atoms will find it more difficult to move. Migration is driven by gradients of force, heat, electricity, and chemical potential, and the effects of these driving forces on different atoms during different stages of migration vary. However, we can confirm that Al atoms are the primary diffusing elements under the influence of the electron wind, while Au atoms can be relatively ignored. This is also the reason why we use the Al element as the main diffusion element when performing element line scanning.

The atomic migration flux caused by electromigration is generally grounded in the theoretical framework of the Huntington and Grone model, then the flux *J*_EM_ can be written as eq S6.^43^ Due to the diffusion value of *D* ^Al^ ≫ *D* ^Au^, which is about 2 orders of magnitude larger, the diffusion flux J_EM_^Al^ caused by electron wind will be significantly greater than J_EM_^Au^.

At the interface of bond 2#, the overall diffusion flux of Al atoms *J*_total_^Al^ (−*J*_EM_^Al^ – *J*_Chem_^Al^) follows the direction of electron flow (assuming the direction of electron flow is negative). The corresponding Au atom flux *J*_total_^Au^, *J*_EM_^Au^ and *J*_Chem_^Au^, both move toward the interface before reaching a steady state during their migration. Under the preferential migration conditions of Al atoms, Au atoms find it challenging to move toward the direction of the electron wind force. Consequently, driven by the conjugate force generated by electromigration, they migrate in the opposite direction. As the Au atoms further migrate and reach the Ti/Ga interface of the barrier layer, they are shielded outside the barrier, accumulating at the interface. Once they reach saturation at the interface, they begin to migrate in the opposite direction. Meanwhile, Au atoms decrease at the interface, falling below saturation. This initiates a repeat cycle, leading to a dynamic equilibrium in atomic flux near the interface for Au atoms.

#### 3.6.3 Current Gathering Effect at the Bond Edge

To investigate the current gathering effect in Au joints at the microscopic scale, ANSYS-FEA was employed to explore the current density during the EM test. It was found that the current density at the edge of the bond is significantly higher than that at the middle, with a difference of 1 order of magnitude between the lowest and highest current densities, as seen in Figure S9b. The Joule effect and electron wind force resulting from the increased current density will further drive the effective charge number, increase the diffusion coefficient, and consequently lead to a greater migration effect at the edge compared to the middle position. The enhanced growth of edge IMC will further intensify this edge effect, which is attributed to the combined influence of current concentration and Joule heating.

#### 3.6.4 Polarity Effect of IMC Growth

For the cathode interface, specifically the 3# bond, the migration of Al and Au atoms following a chemical potential gradient displays the same pattern as in the 2#. The sole distinction arises from the differing electromigration effects influenced by the current direction. Initially, upon electrification, the Al atoms migrate toward the interface at the 3# bond. Similarly, owing to the diffusion barrier layer, Al atoms, similar to Au atoms, experience suppressed diffusion within the barrier layer once they reach the Ti/Ga interface. This interface acts as a diffusion barrier, enabling Al atoms to accumulate continuously. Once the concentration reaches and slightly exceeds the saturation value, Al atoms begin to diffuse away from the interface, following the chemical potential gradient (concentration potential gradient, stress due to atomic stacking, and the temperature-induced higher diffusion coefficient).^44^ The diffusion of Al atoms, driven by electron wind, consistently opposes the ultimate direction of atomic diffusion, ultimately leading to a shorter diffusion distance of Al atoms near the cathode compared with the anode. This clarifies the line scan results observed for the Al element on both the anode and cathode.^45,46^

### 3.7 IMC Grain Evolution during EM

Figure 8 shows the growth and evolution, the propagation of cracks and voids, and the possible mechanism of grain evolution of the IMC at the cathode and anode during EM. The chip-side bond may have undergone the following process. Initial state-1000 h of EM (Figure 8a_1_,a_2_,b_1_,b_2_): (1) EM led to the growth of the IMC layer of the chip-side bond. The IMC was thicker on both sides than in the middle caused by the current gathering effect, which caused the local temperature on both sides to rise. This IMC layer was of moderate thickness and strengthened the bonding. (2) The IMC layer at the anode was slightly thicker and more conducive to crack formation due to the different effects of the comprehensive driving force at the cathode and anode sides. (3) The columnar grains in the middle of the chip-side bond started to enlarge under the action of temperature rise, and enlargements occurred through longitudinal extension and engulfment of small grains.

**Figure 8:**
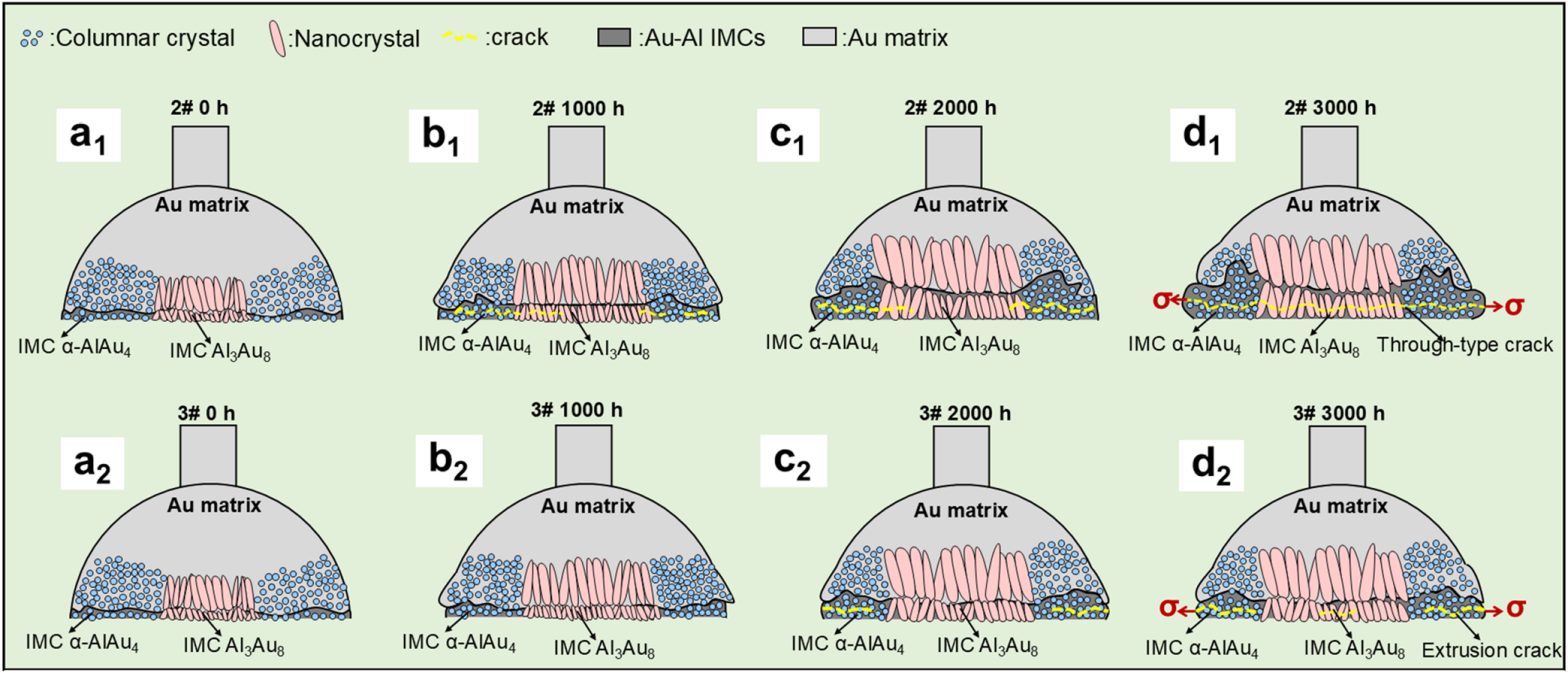
Growth and evolution of cracks and grains in IMC during the EM stages: (a_1_) anode joint EM test 0 h stage, (b_1_) anode joint EM test 1000 h stage, (c_1_) anode joint EM test 2000 h stage, (d_1_) anode joint EM test 3000 h stage, (a_2_) cathode joint EM test 0 h stage, (b_2_) cathode joint EM test 1000 h stage, (c_2_) cathode joint EM test 2000 h stage, and (d_2_) cathode welded joint EM test 3000 h stage.

1000 h–2000 h of EM (Figure 8b_1_,b_2_,c_1_,c_2_): (a) The IMC of the chip-side bond continued to thicken, and the current gathering effect caused the IMC to converge in the bond edge region. At this stage, the IMC thickness exceeded the critical range, leading to the deterioration of the bond’s mechanical properties. (2) The IMC layer at the anode was thicker than that of the cathode, while cracks and voids began to form in the IMC layer at the cathode. (3) The columnar grains in the middle of the chip-side bond were further enlarged.

2000 h–3000 h of EM (Figure 8c_1_,c_2_,d_1_,d_2_): (1) The IMC layer grows excessively and is several times thicker than the critical range. Noticeable cracks appear in the IMC layers on both sides of the joint, accompanied by transverse stress σ on both sides, resulting in a significant deterioration of mechanical properties. (2) The anode IMC layer continued to grow, compressing nearby grains and forming continuous through-cracks. Despite the IMC layer on the cathode overgrowing, it did not compress the nearby grains. (3) Consequently, only small continuous and uniform cracks appeared, while the grain size and orientation distribution of the bond remained essentially unchanged.

## 4 Conclusions

The EM test was conducted at 20 °C on a commercial chip using Au wire bonding with a current density of 10^4^ A/cm^2^. The polarity effect of EM and the structural evolution of the Au–Al interface of the joint were studied. Due to the influence of the polarity effect on the growth and evolution of the IMC layer of the bond, the IMC layer at the interface on both sides of the bond was thicker than the middle interface, and the IMC layer at the interface of the anode joint was thicker than that at the cathode. As the EM test duration increased, the thickness differences in the IMC layer, accentuated by the polarity effect, became more pronounced. The study analyzed the geometric size differences at the Au–Al interface, influenced by temperature gradients, chemical potential gradients, and electron wind forces, from the novel perspective of directional diffusion of metal atoms. The electron wind force produced different influences of the polarity effect at the cathode and anode, promoting Al atom diffusion and migration toward the anode, leading to the IMC phase aggregation at the anode interface, and inhibiting IMC phase growth at the cathode. There were some differences from the previous IMC phase studies on the Au–Al interface. Diffraction and calibration confirmed that the middle region of the joint was a columnar grain Al_3_Au_8_ phase, and both sides of the joint were nanocrystalline α-AlAu_4_ phase. In addition, the morphology of the columnar grains in the core of the joint was highly consistent with that of the Al_3_Au_8_ phase, and the morphology of the nanocrystalline at the edge of the bonding joint was highly consistent with that of the α-AlAu_4_ phase. The above phenomena showed obvious characteristics of grain inheritance. A tensile test was carried out on the interconnection structure of the Au–Al bonding system in commercial chips to evaluate the deterioration of the mechanical properties. Specifically, the failure modes at the cathode and anode evolved from a single neck fracture to detachment of the first joint, with a higher proportion of detachment occurring at the anode than at the cathode. As the duration of the EM test increased, both the maximum average tensile force and the average fracture energy of the first joint initially increased slightly and then decreased sharply. Over the long term, the EM process significantly deteriorated the mechanical properties at the first joint interface.

## Supplementary Material



## Data Availability

The data will be available upon request. All of the figures appearing in the manuscript and Supporting Information file, including figures in the TOC graph, were created by the authors of this manuscript.

## Notes

Structure of the EM test sample; FIB sample preparation process; phase diagram of Au–Al alloy; diffusion distances and growth rates of Al on two polarities joints at different EM stages; TKD phase analysis results for the IMC layer of the complete FIB sample; orientation distribution of each grain in the complete FIB sample; KAM diagram of the distribution of local orientation; KAM diagram of the distribution of local orientation; fracture morphologies at different stages of electromigration; diagram of mass flux and current distribution; equation of the comprehensive driving force *F*; equation of the total atomic flux *J*_total_; equation of the electronic wind power *F*_em_; equation of the effective charge number *Z**; equation of the simplified equation effective charge number *Z**; equation of the atomic migration flux caused by electromigration *J*_EM_; calculation details of the effective charge per atom and the corresponding electronic wind calculation values (PDF)
