# Supplementary material for: Current-Induced Evolving Mechanical Properties, Formation of Defects, and Interfacial Intermetallic Growth in the Interconnects Bonded with Au Wire
Source: ACS Appl Mater Interfaces. 2025 Jun 13;17(25):37193–205. doi: 10.1021/acsami.5c03751 (PMC12203471; doi:10.1021/acsami.5c03751)
Supplement: Supplementary file 1 [file am5c03751_si_001.pdf]

Supporting information

Current induced evolving mechanical property, formation of defects and interfacial intermetallic growth in the interconnects bonded with Au wire

Xiaohong Yuan,[1,*] Qinlian He,[1] Xiaojing Wang,[2,*] Jiaheng Zhang,[2] Dapeng Yang,[2] Qinsong Bi,[1] Yuxi Luo,[1] Dengquan Chen,[1] Shanju Zheng,[3,*] Manal S. Ebaid,[4] Hassan Algadi,[6] and Zhanhu Guo[5,*]

Affiliations

[1] Yunnan Precious Metals Lab, Sino-Platinum Metals Co. Ltd., Kunming, 650106, China

[2] Jiangsu University of Science and Technology, Zhenjiang, 212003, China

[3] Faculty of Material Science and Engineering, Kunming University of Science and Technology, Kunming, 650093, China

[4] Department of Chemistry, College of Science, Northern Border university, Arar, Saudi Arabia

[5] Integrated Composites Lab, Department of Mechanical and Construction Engineering, Northumbria University, Newcastle Upon Tyne, NE1 8ST, UK

[6] Department of Electrical Engineering, Faculty of Engineering, Najran University, Najran, 11001, Saudi Arabia

Corresponding Authors:

Xiaohong Yuan (yxhong87@163.com) ; Xiaojing Wang (wxj@just.edu.cn); Shanju Zheng (zhengshanju1@163.com) or Zhanhu Guo (zhanhu.guo@northumbria.ac.uk)

The file includes:

Supplementary Figure S1-S9

Supplementary Equation S1-S6

Supplementary Calculation details

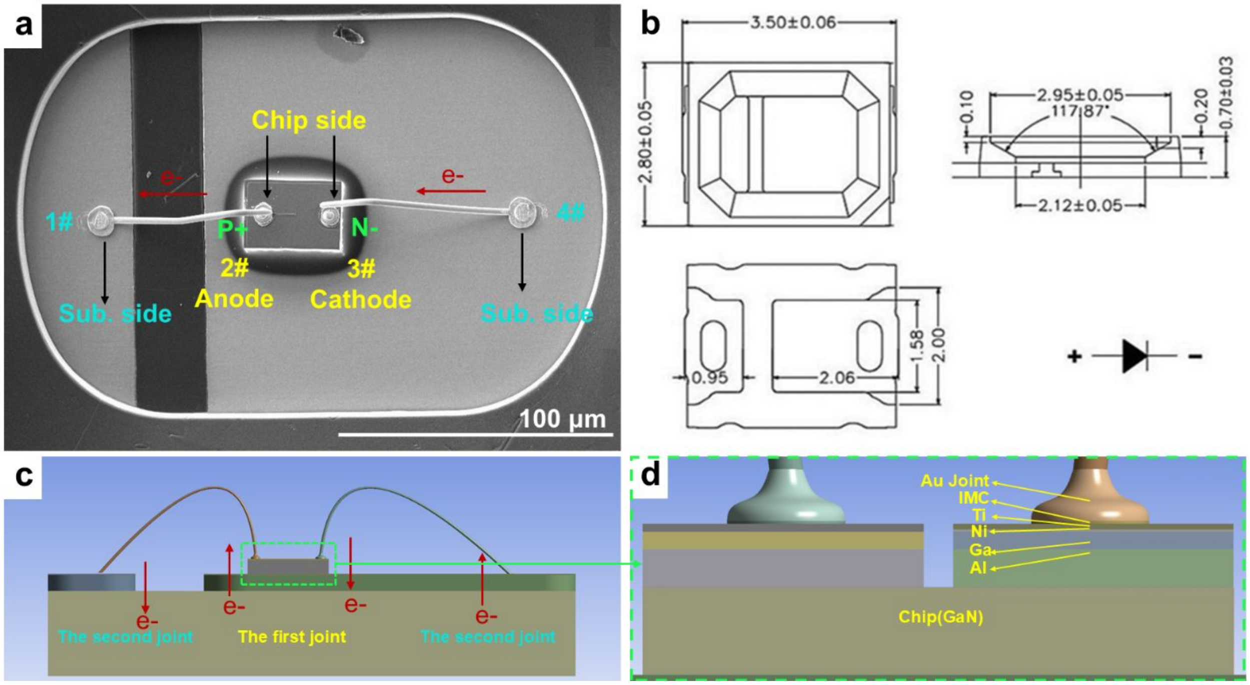

Figure S1. Structure of EM test sample: (a) the diagram of Au-wire bonded chip, (b) the illustration of three-view drawing of Sanan commercial S-118BBMUD model LED chip，(c) the cross-sectional diagram of the bonded chip sample under current-stressing circuit and (d) the cross-sectional diagram of the sample in details.

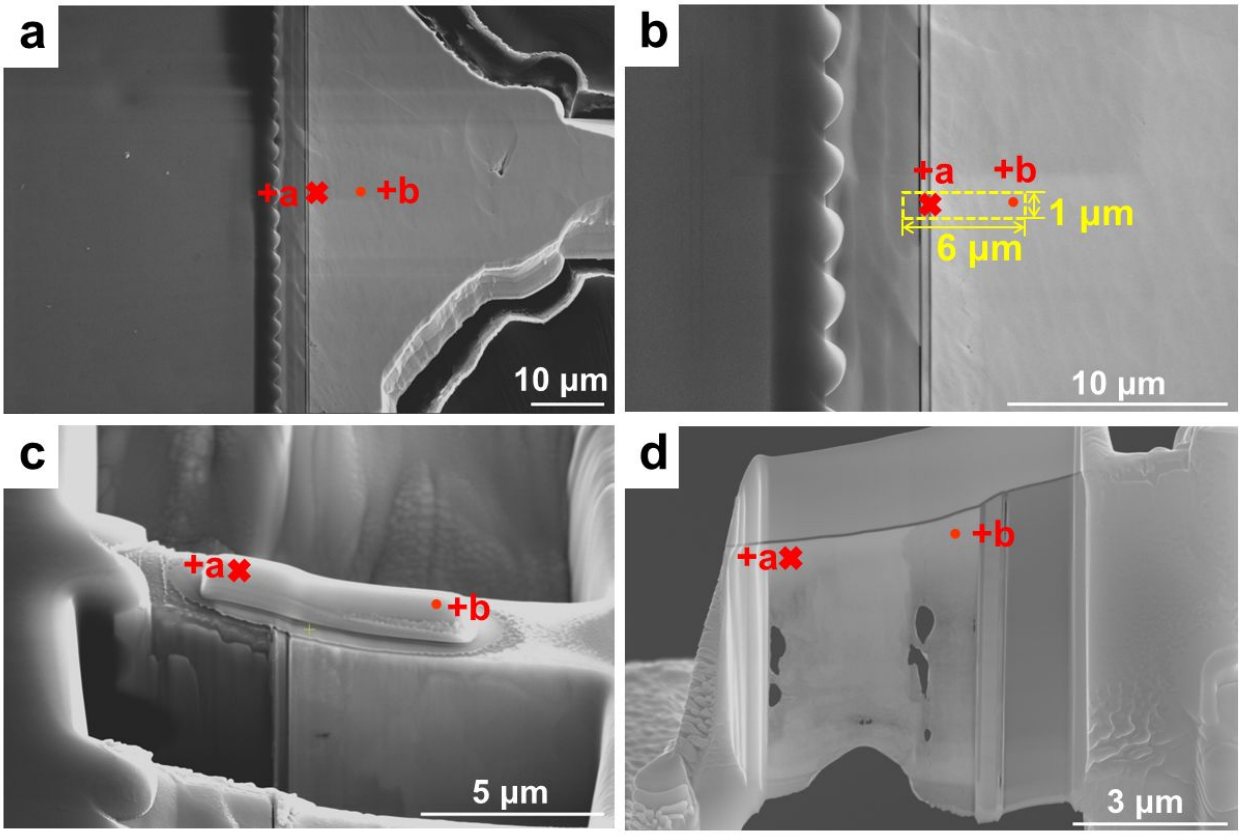

Figure S2. FIB sample preparation process diagram (along the a-d sequence).

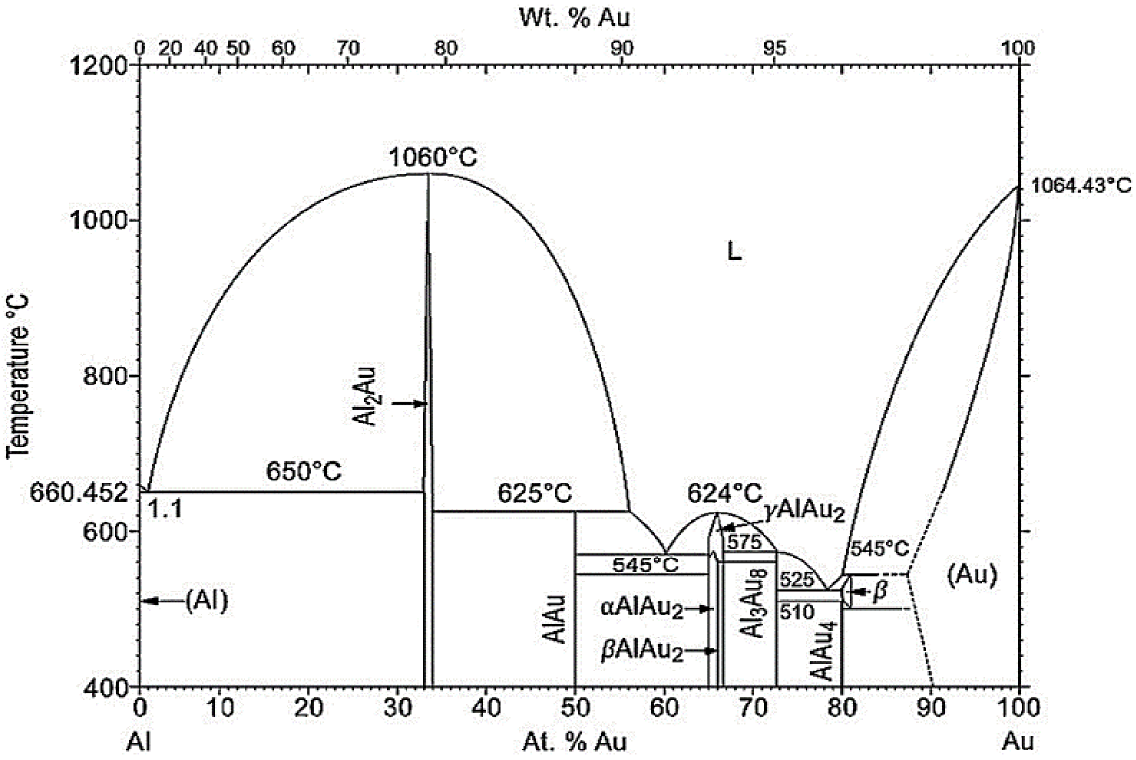

Figure S3. Phase diagram of Al-Au alloy.

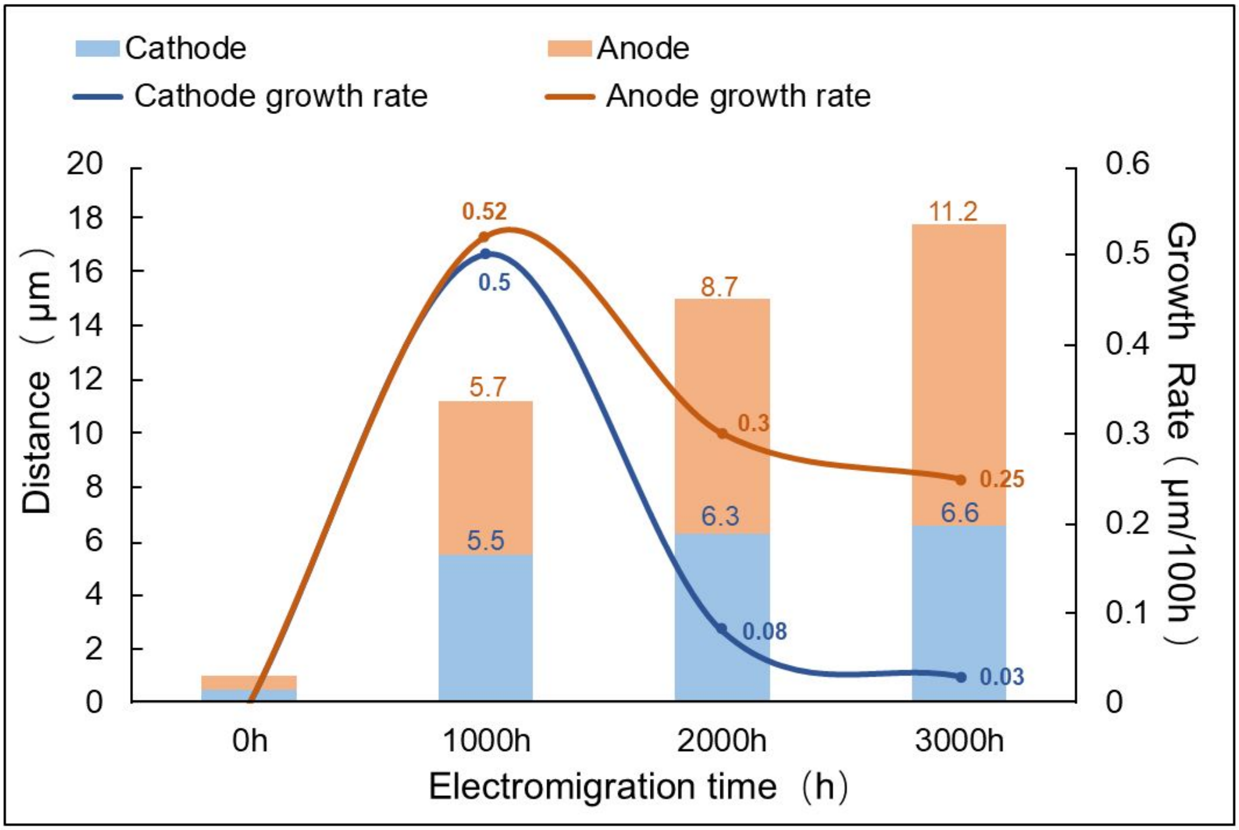

Figure S4. Diffusion distances and growth rates of Al at the cathode and anode at different stages of electromigration.

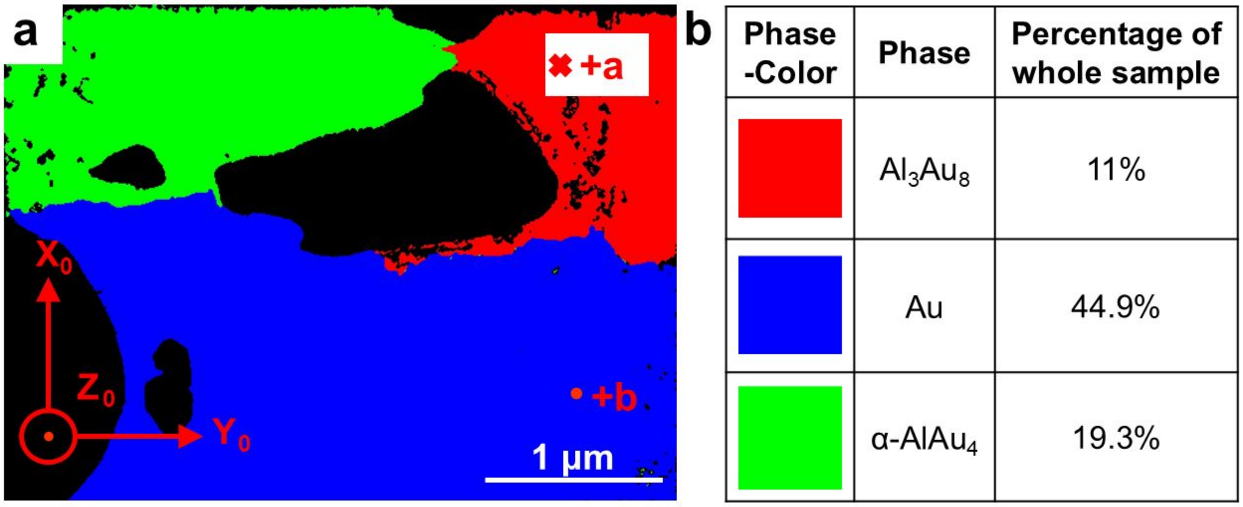

Figure S5. TKD phase analysis results for the IMC layer of the complete FIB sample：(a) Results of the distribution region of each phase in the IMC layer and (b) additional explanation for the TKD results of Figure S5a.

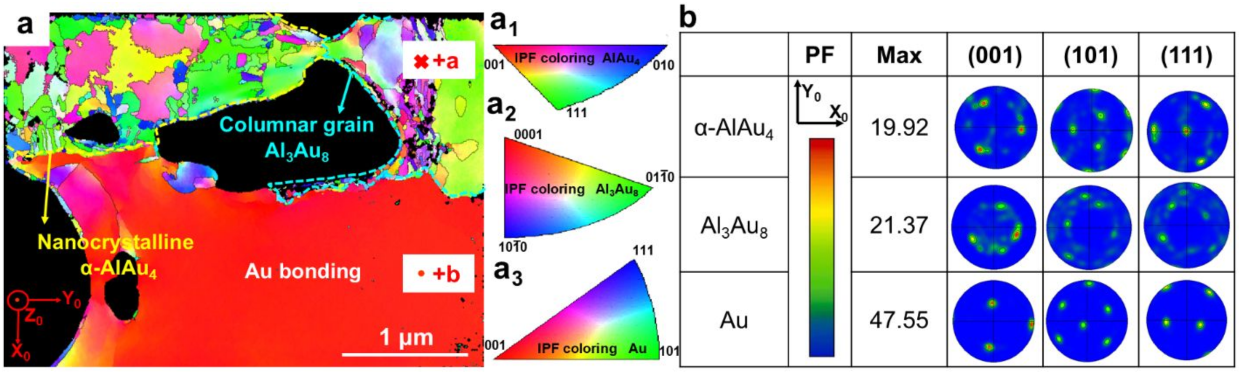

Figure S6. Orientation distribution of each grain in the complete FIB sample: (a) IPF plot along the X_0 direction and (b) PF plots in (001), (101) and (111) directions.

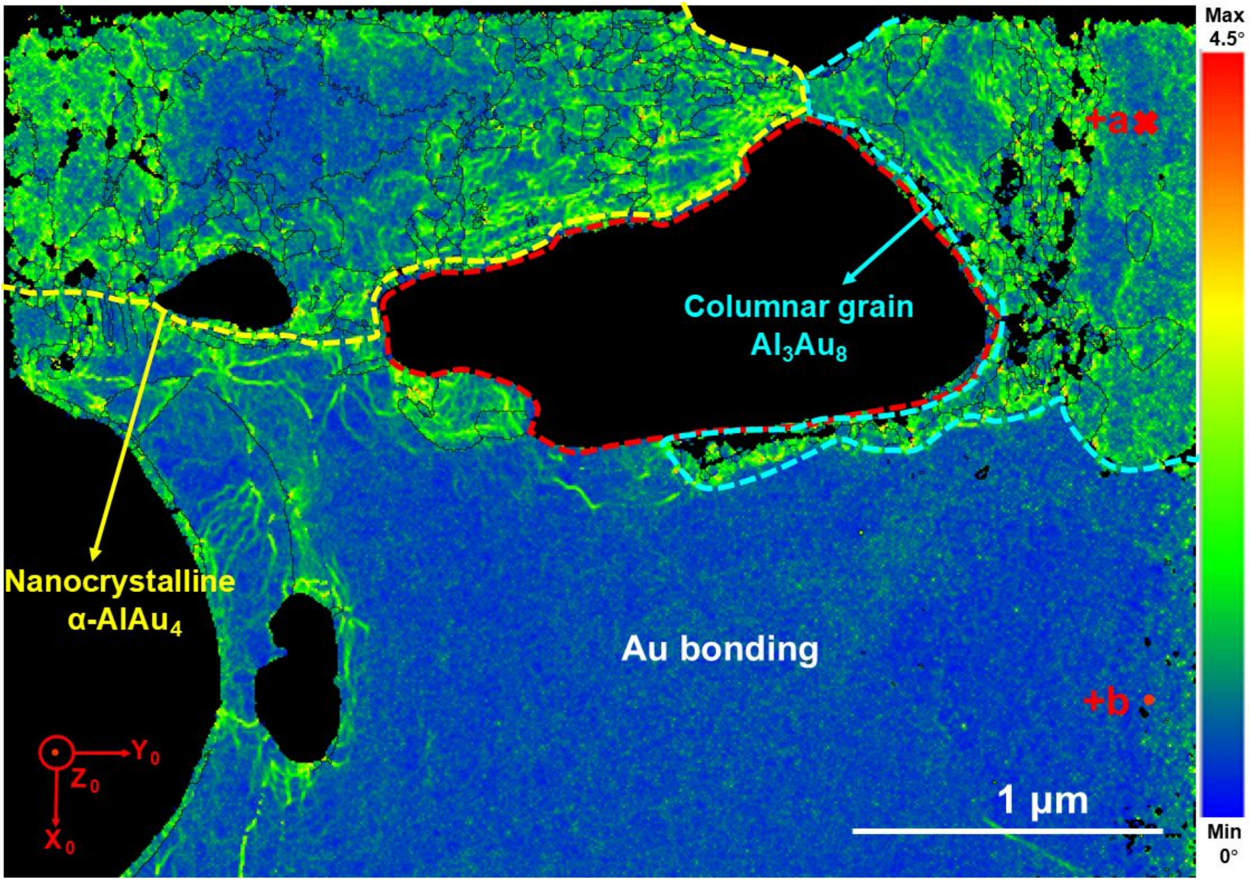

Figure S7. KAM diagram of the distribution of local orientation in the chip side bond after 1000 h of EM.

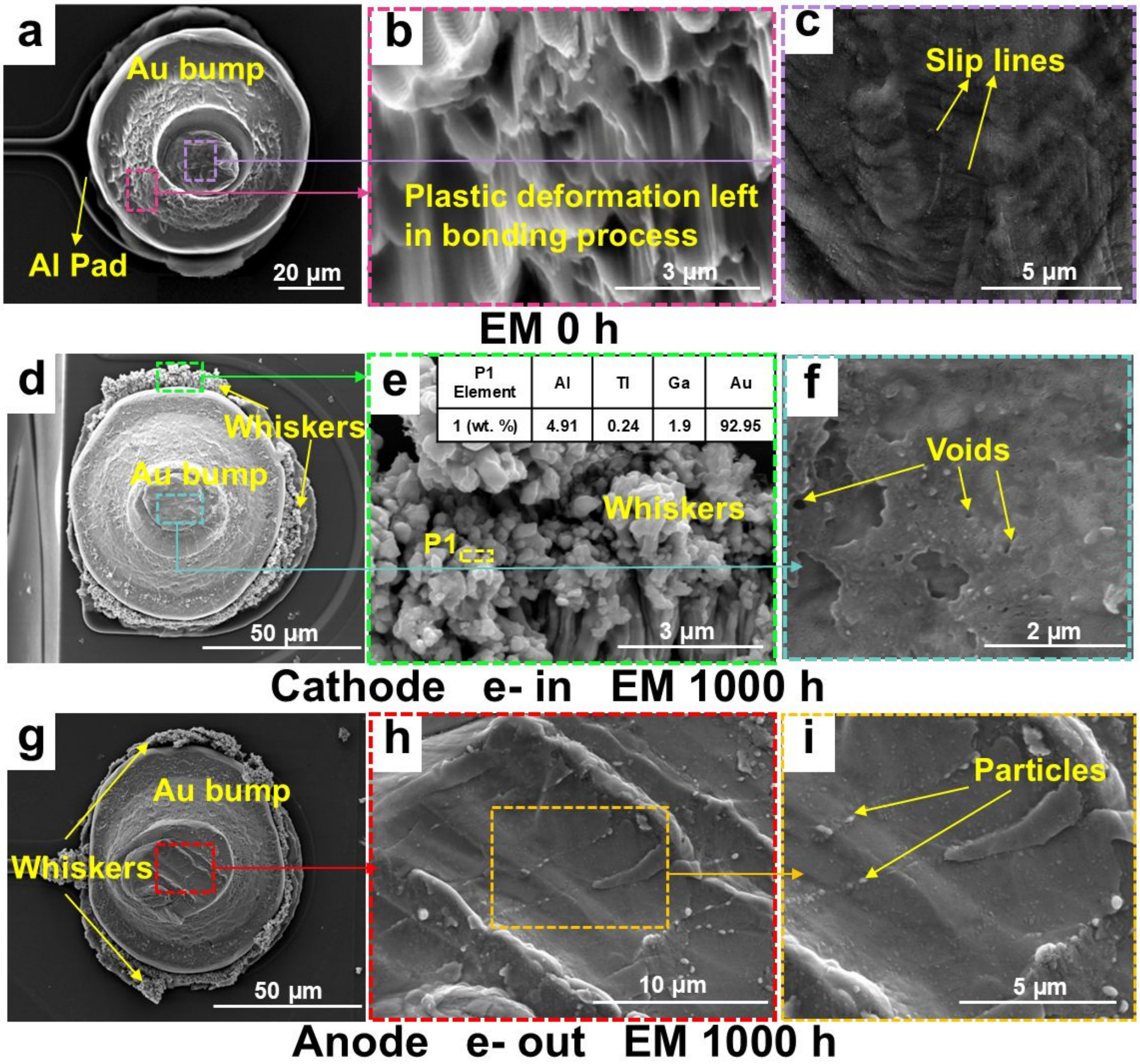

Figure S8. Fracture morphologies: (a)(b)(c) EM 0 h, (d)(e)(f) the cathode and (g)(h)(i) the anode after 1000 h of EM.

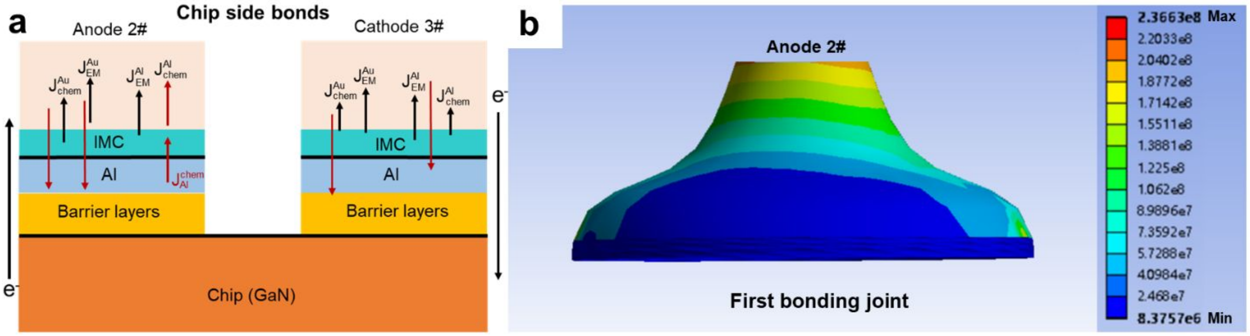

Figure S9. (a) Illustration of mass fluxes within Au wire bonding at cathode and anode sides. (b) Current distribution at the chip side bond during current stressing.

Equation S1:

The comprehensive driving force F:

$$F = F_{em} + F_{\sigma} + F_{\mu} + F_{T}$$

Equation S2:

The Total atomic flux J_{total}:

$$J_{total} = J_{em} + J_{chem}$$

Equation S3:

The electronic wind power F_{em}:

$$F_{em} = Z^{*} e \rho j$$

where Z^{*} is the effective charge number of the atom in electromigration, *e* is the fundamental charge, ρ is the resistivity of the migrating atom, and *j* is the current density.

Equation S4:

The effective charge number Z^{*}:

$$Z^{*} = -Z\left[\frac{\Delta H_{m}}{kT} - 1\right]$$

where *Z* is the nominal valence of the metal atom, ΔH_{m} is the motion activation energy of diffusion, *k* is the Boltzmann constant and *T* is the temperature in Kelvin.

Equation S5:

The simplified equation effective charge number Z^{*}:

$$Z^* = Z - Z\frac{\Delta \mathrm{H}_m}{kT} = Z - \gamma$$

Equation S6:

The atomic migration flux caused by electromigration J_{EM}:

$$J_{EM} = C\frac{D}{kT}F_{em}$$

where *C* is the concentration, *D* is the diffusivity.

Calculation Details:

Effective charge per atom：

$$Z_{\mathrm{Al}}^* = 3(\mathrm{num}) - 3(\mathrm{num}) \times \frac{0.62(\mathrm{eV}) \times 1.602 \times 10^{-19}(\frac{\mathrm{J}}{\mathrm{eV}})}{1.38 \times 10^{-23}\left(\frac{\mathrm{J}}{\mathrm{K}}\right) \times (273+50)\mathrm{K}}$$

$$= 3(\mathrm{num}) - 3(\mathrm{num}) \times \frac{0.993349(\mathrm{J}) \times 10^{-19}}{1.38 \times 10^{-23} \times 323\mathrm{J}} = 3num - 3 \times \frac{7.198 \times 10^3}{(273+T)}\mathrm{num}$$

$$Z_{\mathrm{Al}}^*(50\ \mathrm{oC}) = 3(\mathrm{num}) - 3(\mathrm{num}) \times \frac{7.198 \times 10^3}{(273+50)} = 3(\mathrm{num}) - 3 \times 22.28(\mathrm{num}) = -63.84(\mathrm{num})$$

$$Z_{\mathrm{Au}}^* = 1(\mathrm{num}) - 1(\mathrm{num}) \times \frac{(0.2\sim0.5)(\mathrm{eV}) \times 1.602 \times 10^{-19}\frac{\mathrm{J}}{\mathrm{eV}}}{1.38 \times 10^{-23}\left(\frac{\mathrm{J}}{\mathrm{K}}\right) \times (273+50)\mathrm{K}}$$

$$= 3(\mathrm{num}) - 3(\mathrm{num}) \times \frac{(0.6577\sim1.644) \times 10^{-3}}{10^{-4}}$$

$$= 1(\mathrm{num}) - 1 \times (0.6577\sim1.644) \times 10(\mathrm{num}) = -5.6(\mathrm{num}) - -15.4(\mathrm{num})$$

Then the corresponding electronic wind calculation values are:

$$\mathrm{F}_{\mathrm{EM}}^{\mathrm{Al}} = Z_{\mathrm{Al}}^* e\rho j = -63.84 \times 2.85 \times 10^{-8} ej = -165.8 \times 10^{-8} ej$$

$$\mathrm{F}_{\mathrm{EM}}^{\mathrm{Au}} = Z_{\mathrm{Al}}^* e\rho j = (-5.6\sim-15.4) \times 2.42 \times 10^{-8}\Omega\mathrm{m} ej = (-13.5\sim37) \times 10^{-8} ej$$

$$\frac{\mathrm{F}_{\mathrm{EM}}^{\mathrm{Al}}}{\mathrm{F}_{\mathrm{EM}}^{\mathrm{Au}}} = 4.9\sim13.4$$

Parameters used:

1eV=1.602176634×10^{-19} J

k=1.38 × 10^{-23} J/K

Temperature (°C) =50 °C

Electrical resistivity (Ωm): Au 2.42×10^{-8}; Al2.85×10^{-8} Ωm

Activation energy (eV/atom) [S1]: Au 0.2~0.5; Al :0.62

Reference：

(S1). Tu, K.-N.; Chen, C.; Chen, H.-M. Electronic Packaging Science and Technology; John Wiley & Sons, Inc., 2021.
